# Supplementary material for: Turning the spotlight: Hostile behavior in creative higher education and links to mental health in marginalized groups
Source: PLoS One. 2025 Jan 3;20(1):e0315089. doi: 10.1371/journal.pone.0315089 (PMC11698332; doi:10.1371/journal.pone.0315089)
Supplement: S1 Table — (DOCX) [file pone.0315089.s001.docx]

S 1 Table. Study Subject Clusters within Creative Higher Education According to German Federal Office of Statistics.

| Subject cluster | Subject of study | % of sample |
| --- | --- | --- |
| General art studies |  | 4.9 |
|  | Interdisciplinary studies (focus on art, art science |  |
|  | Art education |  |
|  | Art history, art science |  |
|  | Restoration studies |  |
| Fine arts |  | 19.3 |
|  | Fine arts/graphics |  |
|  | Sculpture/plastics |  |
|  | Painting |  |
|  | New media |  |
| Design studies |  | 21.1 |
|  | Applied arts |  |
|  | Gemstone and jewelry design |  |
|  | Graphic Design/communication design |  |
|  | Industrial design/product design |  |
|  | Textile design |  |
|  | Handicraft education |  |
| Performing arts, film and television, theater studies |  | 8.0 |
|  | Performing arts/stagecraft/directing |  |
|  | Film and television |  |
|  | Acting |  |
|  | Dance education |  |
|  | Theater studies |  |
| Music, musicology |  | 36.0 |
|  | Conducting |  |
|  | Vocal studies |  |
|  | Instrumental music |  |
|  | Jazz and popular music |  |
|  | Sacral music |  |
|  | Composition |  |
|  | Music education |  |
|  | Musicology/history |  |
|  | Orchestral music |  |
|  | Rhythmics |  |
|  | Sound engineering |  |
