# Supplementary material for: Turning the spotlight: Hostile behavior in creative higher education and links to mental health in marginalized groups
Source: PLoS One. 2025 Jan 3;20(1):e0315089. doi: 10.1371/journal.pone.0315089 (PMC11698332; doi:10.1371/journal.pone.0315089)
Supplement: S2 Table — (DOCX) [file pone.0315089.s002.docx]

S 2 Table. Sociodemographic Sample Characteristics.

|  | *n* | % | N |
| --- | --- | --- | --- |
| Gender identity |  |  | 605 |
| Female, trans, inter, non-binary, questioning | 435 | 71.9 |  |
| Male | 161 | 26.6 |  |
| Prefer not to answer | 9 | 1.5 |  |
| Sexual identity |  |  | 600 |
| Lesbian, gay, bisexual, asexual or pansexual | 217 | 36.2 |  |
| Heterosexual | 354 | 59.0 |  |
| Prefer not to answer | 29 | 4.7 |  |
| Care responsibilities |  |  | 597 |
| With care responsibilities | 58 | 9.7 |  |
| Without care responsibilities | 525 | 87.9 |  |
| Prefer not to answer | 14 | 2.3 |  |
| Migration history |  |  | 607 |
| Own and/or at least one parent | 188 | 31.0 |  |
| No migration history | 419 | 69.0 |  |
| Ethnic-racial identity |  |  | 584 |
| Marginalized ethnic-racial identity | 126 | 21.6 |  |
| Non-marginalized ethnic-racial identity | 341 | 58.4 |  |
| Prefer not to answer | 117 | 20.0 |  |
| Mental health issues |  |  | 581 |
| Yes | 244 | 42.0 |  |
| No | 325 | 55.9 |  |
| Prefer not to answer | 12 | 2.1 |  |
| Physical health issues |  |  | 579 |
| Yes | 184 | 31.8 |  |
| No | 379 | 65.5 |  |
| Prefer not to answer | 16 | 2.8 |  |
| Disability |  |  | 580 |
| Yes | 28 | 4.8 |  |
| No | 540 | 93.1 |  |
| Prefer not to answer | 12 | 2.1 |  |
