# Supplementary material for: Turning the spotlight: Hostile behavior in creative higher education and links to mental health in marginalized groups
Source: PLoS One. 2025 Jan 3;20(1):e0315089. doi: 10.1371/journal.pone.0315089 (PMC11698332; doi:10.1371/journal.pone.0315089)
Supplement: S3 Table — (DOCX) [file pone.0315089.s003.docx]

S 3 Table. Experiences of Discrimination.

| Discrimination due to … | *n* mentions | % of *N* = 564 |
| --- | --- | --- |
| …a higher age | 45 | 8.0 |
| …a young age | 35 | 6.2 |
| …gender | 151 | 26.8 |
| …education level | 19 | 3.4 |
| …low income | 55 | 9.8 |
| …care responsibilities | 15 | 2.7 |
| …sexual orientation | 19 | 3.4 |
| …others’ racist attitudes | 52 | 9.2 |
| …religion, philosophy of life | 15 | 2.7 |
| …mental health issues | 45 | 8.0 |
| …disability | 12 | 2.1 |
| …chronic disease | 17 | 3.0 |
| Individual description of discrimination experience | 52 | 9.2 |
| Prefer not to answer | 251 | 44.5 |

*Note*. Multiple response possible.
