# Supplementary material for: Turning the spotlight: Hostile behavior in creative higher education and links to mental health in marginalized groups
Source: PLoS One. 2025 Jan 3;20(1):e0315089. doi: 10.1371/journal.pone.0315089 (PMC11698332; doi:10.1371/journal.pone.0315089)
Supplement: S4 Table — (DOCX) [file pone.0315089.s004.docx]

| Hostile behaviors | | | | | | | | | | | | | | | |
| --- | --- | --- | --- | --- | --- | --- | --- | --- | --- | --- | --- | --- | --- | --- | --- |
|  | Micro aggressions | | | Abuse of power | | | Sexual harassment | | | Sexual violence | | | TFSV | | |
|  | Depr. sympt. | Low WB | Thriving | Depr. sympt. | Low WB | Thriving | Depr. sympt. | Low WB | Thriving | Depr. sympt. | Low WB | Thriving | Depr. sympt. | Low WB | Thriving |
| Variable interacting with respective hostile behavior | *β* | *β* | *β* | *β* | *β* | *β* | *β* | *β* | *β* | *β* | *β* | *β* | *β* | *β* | *β* |
| Gender | .02 | .03 | -.02 | .10 | .14* | -.06 | .06 | .08 | -.03 | -.07 | .04 | .01 | -.03 | -.09 | .02 |
| Sexual orientation | -.01 | -.02 | -.06 | -.14 | -.00 | -.05 | -.13 | -.07 | .00 | -.15 | -.08 | .01 | -.15* | -.08 | .01 |
| Age | -.07 | .01 | .05 | .11 | .01 | .00 | -.01 | -.03 | -.01 | -.02 | -.01 | -.04 | -.02 | .04 | -.05 |
| Care | .05 | .00 | .03 | -.07 | -.04 | -.00 | .00 | .03 | .06 | -.41 | -.13 | .39 | .01 | -.07 | -.41 |
| Migration history | -.17 | .10 | -.15 | .11 | .17 | -.14 | .07 | .05 | -.18 | -.06 | .12 | -.09 | .11 | .21 | -.29 |
| Ethnicity | .25* | .14 | .03 | .09 | -.02 | .15 | -.08 | -.08 | .20 | -.04 | -.07 | .27 | .03 | -.21 | .59* |
| Mental health issues | -.10 | -.02 | .03 | -.05 | -.06 | -.06 | -.24*** | -.16* | .12 | -.01 | .01 | -.05 | -.05 | .05 | -.13 |
| Disability | -.39 | -.60* | -.20 | -.36 | -.49* | -.38 | .24 | .21 | -.34 | -.54 | -.46 | .72 | .10 | .01 | -.19 |
| Physical health issues | -.13* | -.13* | .06 | -.22* | -.20* | .05 | -.15 | -.22* | -.02 | -.18 | .05 | -.13 | -.07 | -.25 | -.11 |

S 4 Table. Moderating Effects of Diversity Domains on Association of Hostile Behavior Experience with Mental Health and Professional Thriving.

*Note*. TFSV: Technology-facilitated sexual violence; Depr.sympt: Depressive symptoms; Low WB: Low well-being.
**p* <.05, *** p* < .01, ****p* <.001; *β*: standardized coefficients*.*
