# Supplementary material for: Turning the spotlight: Hostile behavior in creative higher education and links to mental health in marginalized groups
Source: PLoS One. 2025 Jan 3;20(1):e0315089. doi: 10.1371/journal.pone.0315089 (PMC11698332; doi:10.1371/journal.pone.0315089)
Supplement: S5 Table — (DOCX) [file pone.0315089.s005.docx]

S 5 Table. Micro Aggressions Experience Mediating Association of Diversity Domains with Mental Health, Thriving and Industry Closeness.

| M: Micro aggression experience | | | | | |
| --- | --- | --- | --- | --- | --- |
| IV | UV | DE | IE | Boot LLCI | Boot ULCI |
| Gender identity | Depressive symptoms | -.20** | -.06 | -.11 | -.02 |
|  | Lower well-being | -.19 | -.08 | -.15 | -.02 |
|  | Thriving | .05 | .08 | .03 | .13 |
|  | IOS | .11 | .03 | -.01 | .10 |
| Sexual identity | Depressive symptoms | -.17** | -.02 | -.07 | .02 |
|  | Lower well-being | -.25** | -.04 | -.10 | .02 |
|  | Thriving | .09 | .03 | -.02 | .07 |
|  | IOS | .15 | .01 | -.01 | .04 |
| Age | Depressive symptoms | -.01* | .002 | -.001 | .01 |
|  | Lower well-being | -.001 | .003 | -.001 | .01 |
|  | Thriving | .003 | -.002 | -.005 | .001 |
|  | IOS | -.01 | -.001 | -.004 | .001 |
| Care responsibilities | Depressive symptoms | .01 | -.08 | -.17 | -.002 |
|  | Lower well-being | -.08 | -.12 | -.24 | -.01 |
|  | Thriving | .08 | .08 | -.005 | .18 |
|  | IOS | .21 | .03 | -.03 | .12 |
| Migration history | Depressive symptoms | -.10 | -.04 | -.08 | .01 |
|  | Lower well-being | -.04 | -.05 | -.11 | .02 |
|  | Thriving | .08 | .01 | -.03 | .07 |
|  | IOS | -.26 | .01 | -.02 | .05 |
| Ethnic-racial identity | Depressive symptoms | -.12 | -.07 | -.12 | -.02 |
|  | Lower well-being | -.06 | -.10 | -.18 | -.03 |
|  | Thriving | -.005 | .09 | .03 | .15 |
|  | IOS | -.18 | .05 | -.01 | .15 |
| Mental health issues | Depressive symptoms | -.33*** | -.06 | -.10 | -.02 |
|  | Lower well-being | -.44*** | -.08 | -.15 | -.03 |
|  | Thriving | .23*** | .07 | .03 | .12 |
|  | IOS | .34* | .02 | -.03 | .08 |
| Physical health issues | Depressive symptoms | -.14* | -.04 | -.09 | -.003 |
|  | Lower well-being | -.33*** | -.06 | -.13 | -.01 |
|  | Thriving | .20*** | .05 | .01 | .10 |
|  | IOS | .21 | .02 | -.01 | .07 |
| Disability | Depressive symptoms | -.23 | -.09 | -.21 | .01 |
|  | Lower well-being | -.37 | -.14 | -.30 | .02 |
|  | Thriving | .47*** | .13 | .01 | .28 |
|  | IOS | .40 | .06 | -.03 | .19 |

*Note*. IOS = Inclusion of Other in the Self scale, used to assess closeness to creative industries; IV=independent variable; DV=dependent variable; M=mediator; DE=direct effect; IE=indirect effect; Boot LLCI=bootstrap lower limit confidence interval; Boot ULCI= bootstrap lower limit confidence interval
 **p* <.05 *** p* < .01 ****p* <.001
