# Supplementary material for: Turning the spotlight: Hostile behavior in creative higher education and links to mental health in marginalized groups
Source: PLoS One. 2025 Jan 3;20(1):e0315089. doi: 10.1371/journal.pone.0315089 (PMC11698332; doi:10.1371/journal.pone.0315089)
Supplement: S6 Table — (DOCX) [file pone.0315089.s006.docx]

S 6 Table. Abuse of Power Experience Mediating Association of Diversity Domains with Mental Health, Thriving and Industry Closeness.

| M: Abuse of power experience | | | | | |
| --- | --- | --- | --- | --- | --- |
| IV | UV | DE | IE | Boot LLCI | Boot ULCI |
| Gender identity | Depressive symptoms | -.21** | -.03 | -.06 | -.003 |
|  | Lower well-being | -.22* | -.04 | -.09 | -.004 |
|  | Thriving | .09 | .03 | -.004 | .06 |
|  | IOS | .16 | .00 | -.04 | .04 |
| Sexual identity | Depressive symptoms | -.17* | -.03 | -.06 | -.01 |
|  | Lower well-being | -.23* | -.06 | -.11 | -.02 |
|  | Thriving | .08 | .03 | .01 | .07 |
|  | IOS | .20 | -.01 | -.05 | .04 |
| Age | Depressive symptoms | -.01* | .001 | -.00 | .004 |
|  | Lower well-being | .001 | .003 | -.00 | .01 |
|  | Thriving | .004 | -.002 | -.01 | -.00 |
|  | IOS | -.01 | -.00 | -.00 | .00 |
| Care responsibilities | Depressive symptoms | -.01 | -.03 | -.07 | .01 |
|  | Lower well-being | -.09 | -.05 | -.13 | .01 |
|  | Thriving | .12 | .03 | -.02 | .09 |
|  | IOS | .23 | -.003 | -.05 | .04 |
| Migration history | Depressive symptoms | -.10 | .01 | -.01 | .04 |
|  | Lower well-being | -.04 | .02 | -.02 | .06 |
|  | Thriving | .10 | -.01 | -.05 | .02 |
|  | IOS | -.23 | -.001 | -.03 | .02 |
| Ethnic-racial identity | Depressive symptoms | -.13 | -.02 | -.04 | .01 |
|  | Lower well-being | -.11 | -.03 | -.08 | .02 |
|  | Thriving | .07 | .01 | -.03 | .06 |
|  | IOS | -.09 | .002 | -.02 | .03 |
| Mental health issues | Depressive symptoms | -.37*** | -.02 | -.05 | -.004 |
|  | Lower well-being | -.49*** | -.04 | -.09 | -.01 |
|  | Thriving | .26*** | .03 | .01 | .07 |
|  | IOS | .38* | -.01 | -.06 | .04 |
| Physical health issues | Depressive symptoms | -.15* | -.02 | -.05 | -.001 |
|  | Lower well-being | -.35*** | -.04 | -.08 | -.003 |
|  | Thriving | .22*** | .02 | -.003 | .06 |
|  | IOS | .27 | .001 | -.03 | .04 |
| Disability | Depressive symptoms | -.23 | -.02 | -.07 | .03 |
|  | Lower well-being | -.47* | -.03 | -.13 | .05 |
|  | Thriving | .54*** | .01 | -.05 | .09 |
|  | IOS | .36 | -.001 | -.05 | .04 |

*Note.* IOS = Inclusion of Other in the Self Scale, used to assess closeness to creative industries; IV=independent variable; DV=dependent variable; M=mediator; DE=direct effect; IE=indirect effect; Boot LLCI=bootstrap lower limit confidence interval; Boot ULCI= bootstrap lower limit confidence interval
 **p* <.05 *** p* < .01 ****p* <.001
