# Supplementary material for: Turning the spotlight: Hostile behavior in creative higher education and links to mental health in marginalized groups
Source: PLoS One. 2025 Jan 3;20(1):e0315089. doi: 10.1371/journal.pone.0315089 (PMC11698332; doi:10.1371/journal.pone.0315089)
Supplement: S7 Table — (DOCX) [file pone.0315089.s007.docx]

S 7 Table. Sexual Harassment Experience Mediating Association of Diversity Domains with Mental Health, Thriving and Industry Closeness.

| M: Sexual harassment experience | | | | | |
| --- | --- | --- | --- | --- | --- |
| IV | UV | DE | IE | Boot LLCI | Boot ULCI |
| Gender identity | Depressive symptoms | -.21** | -.02 | -.05 | -.003 |
|  | Lower well-being | -.22* | -.03 | -.07 | -.01 |
|  | Thriving | .09 | .03 | .005 | .06 |
|  | IOS | .19 | -.02 | -.07 | .03 |
| Sexual identity | Depressive symptoms | -.19** | -.01 | -.03 | .005 |
|  | Lower well-being | -.27** | -.02 | -.05 | .004 |
|  | Thriving | .11 | .01 | -.01 | .03 |
|  | IOS | .21 | -.01 | -.04 | .01 |
| Age | Depressive symptoms | -.01* | .001 | .00 | .003 |
|  | Lower well-being | .001 | .002 | .00 | .004 |
|  | Thriving | .004 | -.002 | -.004 | -.00 |
|  | IOS | -.01 | .00 | -.002 | .004 |
| Care responsibilities | Depressive symptoms | .01 | -.03 | -.08 | -.001 |
|  | Lower well-being | -.08 | -.05 | -.11 | -.003 |
|  | Thriving | .11 | .03 | -.01 | .08 |
|  | IOS | .25 | -.01 | -.07 | .03 |
| Migration history | Depressive symptoms | -.09 | .003 | -.02 | .02 |
|  | Lower well-being | -.01 | .01 | -.02 | .03 |
|  | Thriving | .09 | .00 | -.02 | .02 |
|  | IOS | -.22 | .00 | -.02 | .02 |
| Ethnic-racial identity | Depressive symptoms | -.14 | -.01 | -.03 | .01 |
|  | Lower well-being | -.11 | -.01 | -.05 | .01 |
|  | Thriving | .08 | .01 | -.02 | .04 |
|  | IOS | -.07 | -.00 | -.03 | .03 |
| Mental health issues | Depressive symptoms | -.39*** | -.02 | -.04 | .004 |
|  | Lower well-being | -.52*** | -.03 | -.06 | -.00 |
|  | Thriving | .27*** | .03 | .00 | .06 |
|  | IOS | .38* | -.03 | -.09 | .02 |
| Physical health issues | Depressive symptoms | -.16* | -.01 | -.02 | .01 |
|  | Lower well-being | -.38*** | -.01 | -.04 | .02 |
|  | Thriving | .24*** | .004 | -.02 | .03 |
|  | IOS | .27 | -.00 | -.02 | .02 |
| Disability | Depressive symptoms | -.20 | -.05 | -.12 | .003 |
|  | Lower well-being | -.44* | -.06 | -.17 | .005 |
|  | Thriving | .49*** | .06 | .004 | .16 |
|  | IOS | .39 | -.03 | -.19 | .08 |

*Note.* IOS = Inclusion of Other in the Self Scale, used to assess closeness to creative industries; IV=independent variable; DV=dependent variable; M=mediator; DE=direct effect; IE=indirect effect; Boot LLCI=bootstrap lower limit confidence interval; Boot ULCI= bootstrap lower limit confidence interval
 **p* <.05 *** p* < .01 ****p* <.001
