# Supplementary material for: Turning the spotlight: Hostile behavior in creative higher education and links to mental health in marginalized groups
Source: PLoS One. 2025 Jan 3;20(1):e0315089. doi: 10.1371/journal.pone.0315089 (PMC11698332; doi:10.1371/journal.pone.0315089)
Supplement: S8 Table — (DOCX) [file pone.0315089.s008.docx]

S 8 Table. Sexual Aggression Experience Mediating Association of Diversity Domains with Mental Health, Thriving and Industry Closeness.

| M: Sexual aggression experience | | | | | |
| --- | --- | --- | --- | --- | --- |
| IV | UV | DE | IE | Boot LLCI | Boot ULCI |
| Gender identity | Depressive symptoms | -.27*** | -.01 | -.03 | .004 |
|  | Lower well-being | -.28** | -.03 | -.06 | -.002 |
|  | Thriving | .10 | .02 | -.001 | .04 |
|  | IOS | .14 | -.02 | -.06 | .01 |
| Sexual identity | Depressive symptoms | -.22** | -.01 | -.02 | .01 |
|  | Lower well-being | -.26** | -.01 | -.04 | .01 |
|  | Thriving | .11 | .01 | -.01 | .03 |
|  | IOS | .17 | -.01 | -.05 | .01 |
| Age | Depressive symptoms | -.01 | .00 | -.00 | .001 |
|  | Lower well-being | .002 | .00 | -.001 | .002 |
|  | Thriving | .002 | -.00 | -.001 | .00 |
|  | IOS | -.01 | -.00 | -.001 | .001 |
| Care responsibilities | Depressive symptoms | -.05 | -.01 | -.03 | .02 |
|  | Lower well-being | -.25 | -.01 | -.05 | .05 |
|  | Thriving | .14 | .01 | -.01 | .09 |
|  | IOS | .24 | -.01 | -.10 | .03 |
| Migration history | Depressive symptoms | -.14 | -.005 | -.03 | .01 |
|  | Lower well-being | -.04 | -.01 | -.06 | .01 |
|  | Thriving | .09 | .001 | -.02 | .02 |
|  | IOS | -.21 | -.00 | -.02 | .02 |
| Ethnic-racial identity | Depressive symptoms | -.19* | -.005 | -.03 | .01 |
|  | Lower well-being | -.17 | -.01 | -.06 | .01 |
|  | Thriving | .08 | .001 | -.02 | .02 |
|  | IOS | -.08 | -.001 | -.02 | .03 |
| Mental health issues | Depressive symptoms | -.39*** | -.01 | -.02 | .01 |
|  | Lower well-being | -.53*** | -.01 | -.05 | .01 |
|  | Thriving | .28*** | .01 | -.002 | .04 |
|  | IOS | .37** | -.01 | -.05 | .01 |
| Physical health issues | Depressive symptoms | -.17* | -.001 | -.01 | .02 |
|  | Lower well-being | -.39*** | -.002 | -.02 | .03 |
|  | Thriving | .24*** | .001 | -.01 | .03 |
|  | IOS | .25 | -.001 | -.02 | .03 |
| Disability | Depressive symptoms | -.35* | -.03 | -.08 | .02 |
|  | Lower well-being | -.65** | -.06 | -.14 | .04 |
|  | Thriving | .52*** | .04 | -.01 | .18 |
|  | IOS | .42 | -.06 | -.27 | .04 |

*Note.* IOS = Inclusion of Other in the Self Scale, used to assess closeness to creative industries; IV=independent variable; DV=dependent variable; M=mediator; DE=direct effect; IE=indirect effect; Boot LLCI=bootstrap lower limit confidence interval; Boot ULCI= bootstrap lower limit confidence interval
 **p* <.05 *** p* < .01 ****p* <.001
