# Supplementary material for: Turning the spotlight: Hostile behavior in creative higher education and links to mental health in marginalized groups
Source: PLoS One. 2025 Jan 3;20(1):e0315089. doi: 10.1371/journal.pone.0315089 (PMC11698332; doi:10.1371/journal.pone.0315089)
Supplement: S9 Table — (DOCX) [file pone.0315089.s009.docx]

S 9 Table. Technology-Facilitated Sexual Violence Experience Mediating Association of Diversity Domains with Mental Health, Thriving and Industry Closeness.

| M: Technology-facilitated sexual violence experience | | | | | |
| --- | --- | --- | --- | --- | --- |
| IV | UV | DE | IE | Boot LLCI | Boot ULCI |
| Gender identity | Depressive symptoms | -.30*** | -.01 | -.03 | .01 |
|  | Lower well-being | -.33** | -.01 | -.03 | .02 |
|  | Thriving | .13 | .01 | -.01 | .03 |
|  | IOS | .15 | -.02 | -.05 | .01 |
| Sexual identity | Depressive symptoms | -.21** | -.02 | -.05 | -.003 |
|  | Lower well-being | -.26** | -.02 | -.06 | .01 |
|  | Thriving | .10 | .03 | .00 | .06 |
|  | IOS | .22 | -.05 | -.10 | -.002 |
| Age | Depressive symptoms | -.01 | -.00 | -.002 | .00 |
|  | Lower well-being | .002 | -.00 | -.004 | .00 |
|  | Thriving | .002 | .00 | .00 | .002 |
|  | IOS | -.01 | -.001 | -.003 | .00 |
| Care responsibilities | Depressive symptoms | -.05 | .03 | -.06 | .02 |
|  | Lower well-being | -.24 | -.02 | -.07 | .03 |
|  | Thriving | .08 | .03 | -.02 | .14 |
|  | IOS | .28 | -.04 | -.16 | .04 |
| Migration history | Depressive symptoms | -.12 | -.00 | -.03 | .01 |
|  | Lower well-being | -.03 | .001 | -.03 | .03 |
|  | Thriving | .09 | -.003 | -.03 | .01 |
|  | IOS | -.23 | .005 | -.03 | .03 |
| Ethnic-racial identity | Depressive symptoms | -.15 | -.003 | -.04 | .01 |
|  | Lower well-being | -.13 | -.003 | -.05 | .02 |
|  | Thriving | .09 | .00 | -.03 | .02 |
|  | IOS | -.10 | -.00 | -.05 | .03 |
| Mental health issues | Depressive symptoms | -.37*** | -.001 | -.02 | .01 |
|  | Lower well-being | -.50*** | -.001 | -.03 | .02 |
|  | Thriving | .28*** | .01 | -.004 | .03 |
|  | IOS | .37* | -.02 | -.06 | .01 |
| Physical health issues | Depressive symptoms | -.17* | -.002 | -.01 | .03 |
|  | Lower well-being | -.38*** | -.003 | -.02 | .04 |
|  | Thriving | .23*** | .003 | -.01 | .03 |
|  | IOS | .25 | -.004 | -.04 | .03 |
| Disability | Depressive symptoms | -.35* | -.06 | -.13 | .03 |
|  | Lower well-being | -.67** | -.06 | -.15 | .07 |
|  | Thriving | .43** | .07 | -.02 | .27 |
|  | IOS | .53 | -.13 | -.44 | .04 |

*Note.* IOS = Inclusion of Other in the Self Scale, used to assess closeness to creative industries; IV=independent variable; DV=dependent variable; M=mediator; DE=direct effect; IE=indirect effect; Boot LLCI=bootstrap lower limit confidence interval; Boot ULCI= bootstrap lower limit confidence interval
*p* <.05 *** p* < .01 ****p* <.001
